# Can Artificial Intelligence Help Us in the Evaluation of Coronary Artery Calcification Scores by Acting as a Prognosticator in Patients That Are Operated on Due to Non-Small Cell Lung Cancer? A Pivotal Study

**DOI:** 10.3390/jcm13216579

**Published:** 2024-11-01

**Authors:** Tomasz Marjanski, Michal Chmielecki, Kaja Klein-Awerjanow, Wojciech Cytawa, Patrycja Ciepialowska, Andrii Bilyk, Rafal Peksa, Magdalena Dudek

**Affiliations:** 1 Department of Thoracic Surgery, Faculty of Medicine, Medical University of Gdansk, 80-214 Gdansk, Poland; 2 Department of Cardiology, Faculty of Medicine, Medical University of Gdansk, 80-214 Gdansk, Poland; 3 Department of Radiology, Faculty of Medicine, Medical University of Gdansk, 80-214 Gdansk, Poland; 4 Department of Nuclear Medicine, Faculty of Health Sciences, Medical University of Gdansk, 80-214 Gdansk, Poland; 5 Faculty of Medicine, Medical University of Gdansk, 80-214 Gdansk, Poland; 6 Department of Pathology, Faculty of Medicine, Medical University of Gdansk, 80-214 Gdansk, Poland; 7 1st Department of Cardiology, Poznan University of Medical Sciences, 61-701 Poznan, Poland

**Keywords:** lung cancer, surgery, complication, coronary artery disease, CAC score, artificial intelligence

## Abstract

**Background:** Non-small cell lung cancer (NSCLC) is the leading cause of death from malignancies, and surgical resection is the most effective form of treatment. Coronary artery disease (CAD) is a common comorbidity in patients with NSCLC. A coronary artery calcium (CAC) score correlates with the extent of CAD. We aimed to test whether an automated assessment of CAC scores helps to identify the population of patients with a higher risk of postoperative complications and worse overall survival (OS) after the surgical treatment of NSCLC. **Methods:** In this retrospective cohort study, the data of the patients who were surgically treated for NSCLC were matched with the reassessed preoperative CT images. The postoperative complication rates and overall survival were analyzed. The CAC score was evaluated automatically using the Syngo.via Siemens Healthcare software. Cardiac age was assessed according to Hoff et al. 2001. The prognosticators of postoperative complications and of OS were tested. **Results:** The data of 193 patients with complete data, an adherence to the inclusion and exclusion criteria, and that were operated between 2018 and 2019, were included. Cardiac age was a predictor of the cardiovascular and pulmonary complications rate (95%CI −0.007–0.203, *p* = 0.066, beta coefficient 0.098). In a multivariable stepwise regression analysis, operative access was a predictor of cardiovascular and pulmonary complications (95%CI −0.290–−0.111, *p* < 0.001, beta coefficient −0.200), cardiovascular complications (95%CI −0.161–−0.022, *p* = 0.011, beta coefficient −0.036), and the general complication rate (95%CI −0.370–−0.194, *p* < 0.001, beta coefficient −0.286). Kaplan–Meier curves were separated in the survival analysis of groups of patients with a cardiac age 0–69 years vs. an age of 70+ (92 vs. 92 patients) (in Cox regression analysis, HR = 1.678, 95%CI 0.847–3.292 *p* = 0.138). **Conclusions:** An automated CAC score assessment may be a potential and clinically meaningful prognosticator of both postoperative complications and OS in patients that are operated on due to NSCLC. Further studies are required.

## 1. Background

Non-small cell lung cancer (NSCLC) is the leading cause of death from malignancies in both men and women populations [1]. The most efficient way to treat patients with early lung cancer is surgical resection [2]. The surgical treatment of lung cancer requires careful risk assessment, as there are treatment alternatives in the case of high-risk patients [3]. Despite the use of existing protocols of physiologic evaluation [4] and the need to offer the lowest-risk treatment options, there are failures that have been highlighted that lead to postoperative complications and deteriorated overall survival. Diagnosed comorbidities influence the risk assessment process [5]. However, coexisting diseases in the asymptomatic period can confound even the most careful qualification process. One of the most important comorbidities in patients with lung cancer is coronary artery disease (CAD), due to the sharing of a common risk factor—smoking [6]. The correlation between coronary artery calcification (CAC) and CAD is well-documented. In patients who are asymptomatic and do not have confirmed CAD, the detection of CAC through computed tomography (CT) scans serves as a reliable indicator of underlying atherosclerosis [7]. All NSCLC patients who are operated on have their CT carried out in the preoperative period, but the CAC score is not routinely assessed at this level. This raises a question: does coronary artery disease diagnosed by means of a coronary artery calcification score assessment in CT have an impact on perioperative risk and the long-term results of surgical treatment in patients with non-small cell lung cancer?

## 2. Material and Methods

### 2.1. Study Population

**Figure 1:**
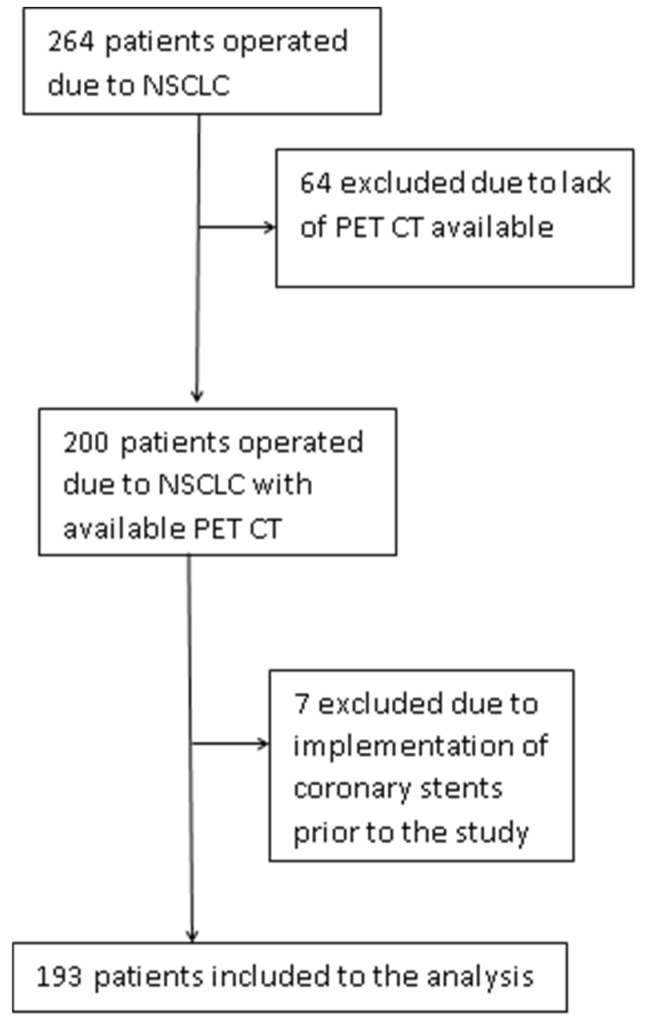
Study flowchart showing the selection of patients. NSCLC, non-small cell lung cancer; PET CT, positron emission tomograph computerized tomography.

In this retrospective cohort study, the data of the patients who were surgically treated for NSCLC in the Thoracic Surgery Department Medical University of Gdansk, Poland were matched with the reassessed preoperative CT images. The postoperative complication rates and overall survival were analyzed. The clinical data were extracted from the KBRP (Krajowa Baza Raka Pluca—National Registry of Lung Cancer). The data in the KBRP are collected prospectively. The data of every consenting patient treated surgically due to NSCLC in Poland are included in this obligatory database. The data were made available for the contributing researchers. The CTs for CAC evaluation were extracted from the Positron Emission Tomography Computed Tomography (PET CT) images and downloaded from the UCK-PACS radiological server. The overall survival data were obtained from the KBRP. The data of 264 patients that were operated on between 4 January 2018 and 6 March 2019 were included. Finally, 193 patients who were operated on due to NSCLC had their PET CT performed preoperatively, and the scans were made available for the automated CAC analysis in this study. The study flowchart illustrating the inclusion criteria for the study is presented in Figure 1. The data were stored in the anonymized version to avoid violating the General Data Protection Regulation. This analysis was approved by the Ethics Committee of the Medical University of Gdansk, which waived individual patient consent due to the observational character of the study.

### 2.2. Endpoints

The primary outcome measures were as follows: the cardiovascular complication rate in the postoperative period, the cardiovascular and pulmonary complication rates, the general complication rate, and overall survival. The secondary outcome measures were as follows: the general complication rate in the postoperative period, the cut-off value of the CAC score differentiating groups with different complication rates, and the different overall survival rates. The issues were noted down in health and care records, which served as the primary source for the national database. The general complications were noted and included: bleeding that needed another operation, hematoma that needed another operation, the requirement for the transfusion of 2 or more units of blood, an early bronchial stump fistula, persistent pneumothorax, pneumothorax that needed redrainage, infection in the wound, pleural empyema without fistula, an air leak lasting more than 7 days, late bronchial stump fistula, urinary tract infection, other infections, kidney insufficiency, chylothorax, other issues that needed another operation, other issues that were treated conservatively, and the paresis of recurrent laryngeal nerve.

For the purpose of analysis, we pulled out a set of combined cardiovascular and pulmonary complications that were also reported in the database: respiratory insufficiency that required reintubation, an atrial arrhythmia that required treatment, ventricular arrhythmia that required treatment, atelectasis that required bronchial aspiration, extended mechanical ventilation, pneumonia, adult respiratory distress syndrome, respiratory insufficiency that required tracheostomy, extended mechanical ventilation, myocardial infarction, pulmonary emboli, other respiratory complications, thrombophlebitis, and other heart-related complications. The cardiovascular complications included the following: an atrial arrhythmia that required treatment, ventricular arrhythmia that required treatment, myocardial infarction, pulmonary emboli, heart-related complications, cerebral infarction, and other neurological complications.

### 2.3. Eligibility Criteria

The eligibility criteria were as follows: 18 years or older, men and women. The inclusion criteria included patients treated surgically due to NSCLC in Thoracic Surgery at the Department Medical University of Gdansk, patients with PET CT performed in the Medical University of Gdansk with CT enabling a CAC score assessment, patients that had their clinical data input in the database KBRP (Krajowa Baza Raka Pluca—National Registry of Lung Cancer), an Eastern Cooperative Oncology Group (ECOG) performance status of 0 or 1, confirmed histologically pathological stage I or II, IIIA, or Select IIIB (T3N2 only) according to the 8th edition of the AJCC/UICC staging system, R0 resection with curative intent by an attending thoracic surgeon, a sufficient pulmonary and cardiac function for surgical resection, and an adequate hematologic and end-organ function. The exclusion criteria included the following: patients with a coronary stent or other conditions, excluding appropriate automated coronary arteries assessment.

### 2.4. Radiological Assessment

The CT protocol was as follows: The patients were injected with an activity of 3.3 MBq of 18F-FDG per kg of body weight. Imaging was performed exclusively with a 40-detector PET/CT scanner (Siemens Biograph mCT 40, Siemens Healthineers, Erlangen, Germany) after an uptake period of ~60 min. PET emission data were acquired from 6 to 8 bed positions (depending on the patient’s height), from the base of the skull to the proximal thighs (2 min emission time per bed position for <90 kg of body weight, 3 min for 90–120 kg, and 4 min for ≥120 kg patients). Subsequently, a monophasic low-dose CT scan without contrast media was performed (CARE Dose 4D, 160 mAs, 120 kV, 512 × 512 matrix, 2 mm slice thickness, slice collimation 64 × 0.6 mm, pitch index: 1.25). All PET emission data were reconstructed with an iterative algorithm (TrueX + TOF, ultra HD-PET, 21 subsets, 2 iterations, Gaussian filtering: 4.5 mm, Zoom 1.0, 171 × 200 × 200 matrix, axial resolution: 5 mm, in-plane resolution: 4.07 × 4.07 mm^2^) using dedicated manufacturer software (syngo MI.PET/CT VG62C, Siemens Healthineers, Erlangen, Germany). No adverse events, including allergic reactions, were observed after the administration of radiotracer.

### 2.5. CAC Scoring Assessment

The CAC score was evaluated automatically by the principal investigator (TM) using the Syngo.via Siemens Healthcare VB60S_HF1 (Malvern, PA, USA) software. The measurements were performed utilizing the Agatson CAC score algorithm. The exact result of the CAC score was assessed on the basis of the Ca-Score function of the Syngo.via platform. In the paper by Hoff et al., coronary age was assessed using electron beam tomography [8]. The study involved 35,246 asymptomatic individuals aged 30 to 90 years. The CAC score, which reflected the amount of calcium in the coronary arteries, was calculated based on the number, area, and peak density of calcific lesions detected. The researchers developed age- and gender-specific distributions of CAC scores to serve as standards for clinical interpretation. These distributions allowed a comparison of an individual’s CAC score with those of others in the same age and gender group, providing a percentile ranking. This ranking allowed an estimation of the “coronary age” of a patient, which can be higher or lower than their chronological age depending on their CAC score. The data concerning the total CAC score, coronary age, and CAC score of coronary arteries were aggregated.

### 2.6. Statistical Analysis

Unpaired data, following a normal distribution, were compared using an unpaired *t*-test, while a Mann–Whitney U-test was employed for non-normally distributed variables to compare two unmatched samples. The χ^2^ test was utilized for categorical variables, with a set significance level of *p* = 0.05. Odds ratios (ORs) with 95% confidence intervals (CIs) were calculated.

We created 6 models of multivariable logistic regression using the following dependent variables: general complications rate, cardiovascular complications rate, and cardiovascular and pulmonary complications rate. In the first three models, we used the following predictors: FEV1%, FVC%, surgical access (VATS vs. thoracotomy), extent of surgery (lobectomy vs. segmentectomy vs. pneumonectomy), CAC score, and cardiac age. The second set of predictors included the CAC score, the scores of the left main coronary artery (LM), left anterior descending coronary artery (LAD), left circumflex coronary artery (CX), and the right coronary artery (RCA). We used conservative *p* value 0.2 to exclude factors in the stepwise regression analysis.

The automated assessment of the coronary age was recorded and validated in the study cohort.

The patients were assigned into different groups based on their CAC score values to test the threshold value differentiating groups with different overall survival, i.e., 0–50 vs. 51+, 0–100 vs. 101+, 0–300 vs. 301+, 0–500 vs. 501+, 0–1000 vs. 1001+, and 0–2000 vs. 2001+. In order to validate the effect of cardiac age on the overall survival, patients were stratified into 0–29 vs. 30+, 0–44 vs. 45+, 0–49 vs. 50+, 0–49 vs. 50+, 0–54 vs. 55+, 0–59 vs. 60+, 0–64 vs. 65+, 0–69 vs. 70+, 0–74 vs. 75+, and 0–89 vs. 90+. Survival probability was evaluated by the Kaplan–Meier method.

Statistical analysis was performed with the Stata 18.0, BE—Basic Edition, StataCorp LLC, StataCorp, 4905 Lakeway Drive, College Station, TX 77845, USA.

The University Ethics Committee approved the study, which waived the individual consent due to the study’s retrospective nature.

## 3. Results

A total of 264 patients were enrolled and 193 finally entered the statistical analysis. The study flowchart is presented in Figure 1.

The patients’ characteristics are presented in Table 1.

**Figure 2:**
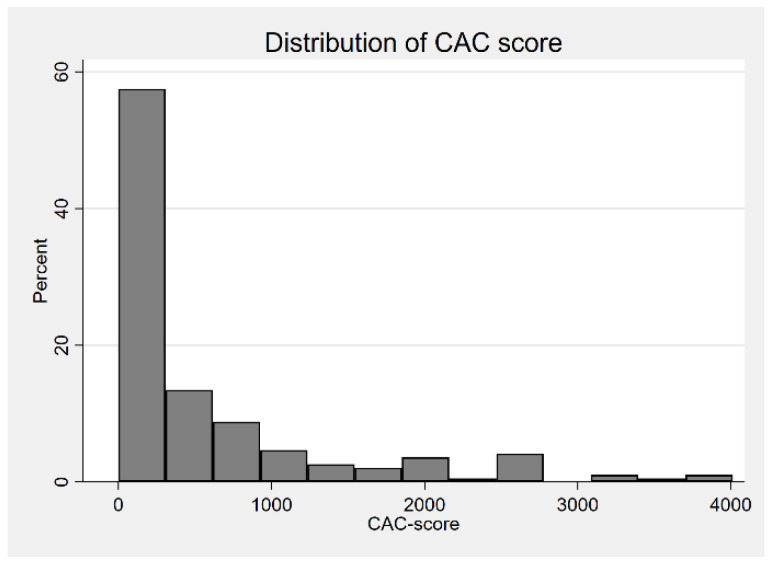
Histogram of CAC score. CAC, coronary artery score.

**Figure 3:**
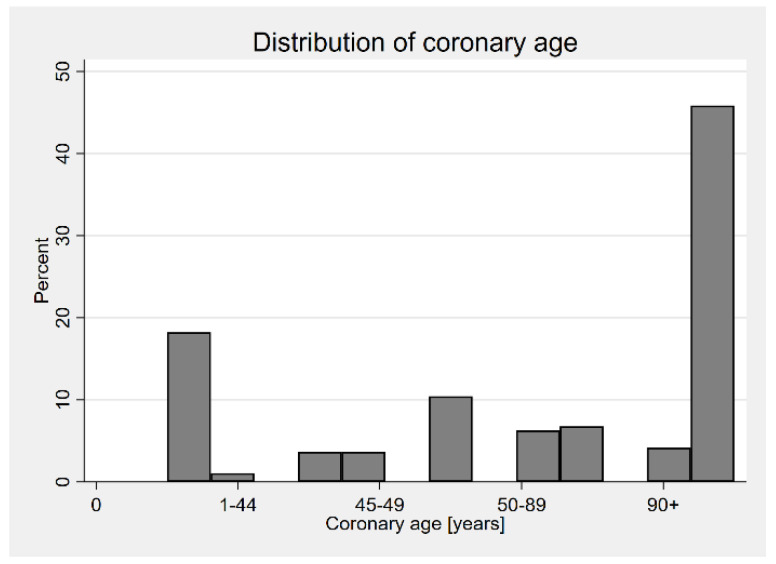
Distribution of coronary age according to Hoff et al [8].

The histograms concerning the distribution of the CAC score and the coronary age are presented in Figure 2 and Figure 3.

The CAC scores and assessed coronary age are presented in Table 2. The complication rates are presented in Table 3. The follow-up was assessed for every patient, with a median period of observation of 49 months.

There was a trend in the logistic regression analysis showing that the cardiac age was a predictor of cardiovascular and pulmonary complications rate (95%CI −0.007–0.203, *p* = 0.066, beta coefficient 0.098). The VATS approach was related to a reduced occurrence of general complications (95%CI −1.288–−0.028, *p* = 0.041, beta coefficient −0.658). The Age-adjusted Charlson Comorbidity Index was an independent predictor of the occurrence of cardiovascular complications (95%CI −0.010–0.478, *p* = 0.041, beta coefficient 0.244). Other factors did not influence the occurrence of the three sets of postoperative complications.

In the multivariable stepwise regression analysis, operative access (95%CI −0.290–−0.111, *p* < 0.001, beta coefficient −0.200) was a predictor of cardiovascular and pulmonary complications, as well as cardiovascular complications (95%CI −0.161–−0.022, *p* = 0.011, beta coefficient −0.036) and the general complication rate (95%CI −0.370–−0.194, *p* < 0.001, beta coefficient −0.286). The models are presented in Table 4a–f.

**Figure 4:**
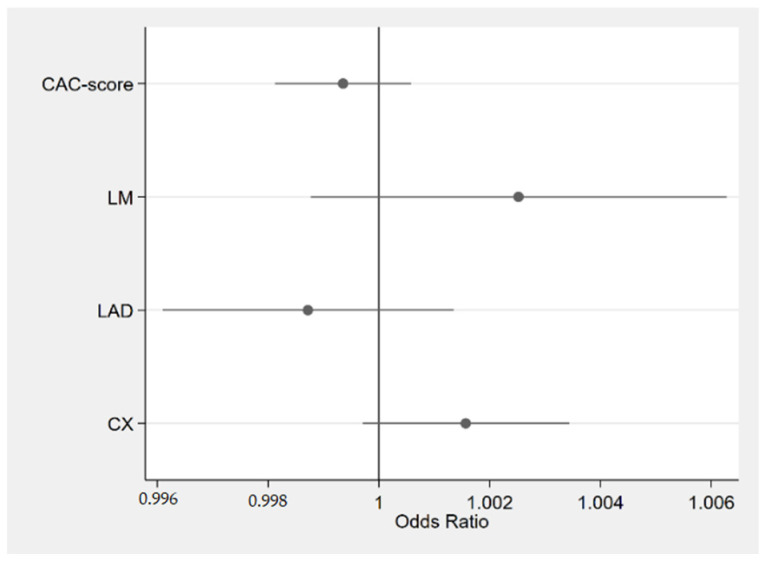
Forest plot of odds ratios of CAC score and calcifications in other coronary arteries. Horizontal lines represent 95% confidence interval. CAC, coronary artery cardiac score; LM, left main coronary artery; LAD, left anterior descending coronary artery; CX, left circumflex coronary artery.

In this relatively small group, there were no prognosticators that significantly influenced the OS. However, the analysis of the trends in the groups revealed that the higher that the CAC score and coronary age were diagnosed, the worse the OS was in most of the analyses. The results in the analysis of the scores of calcifications in individual coronary arteries were less unequivocal (Figure 4).

**Figure 5:**
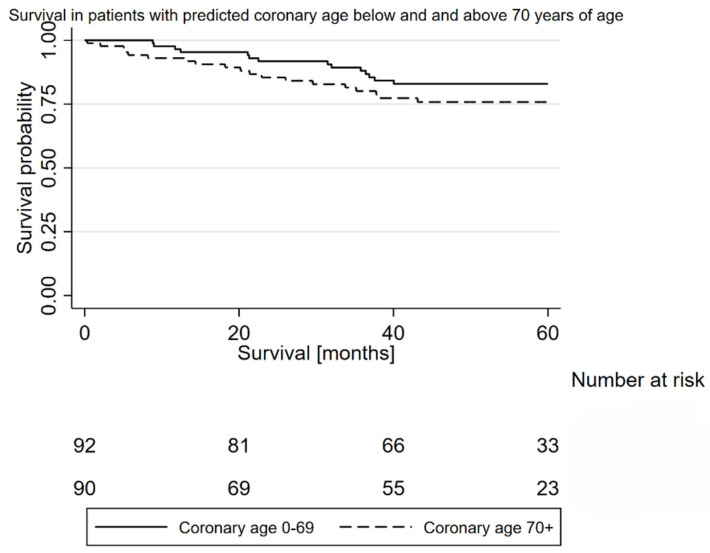
Kaplan–Meier curves illustrating overall survival in patients in coronary ages 0–69 and 70+. HR = 1.67, 95%CI 0.847–3.292 *p* = 0.138.

No opposite trends were observed. The Kaplan–Meier curves were the most significantly separated in the case of the survival analysis in the two groups of patients with cardiac ages pf 0–69 years of age and 70+ (92 vs. 92 patients). In the log rank test, the *p* = 0.137. In the Cox regression analysis, the HR = 1.678, 95%CI 0.847–3.292 *p* = 0.138 (Figure 5).

Finally, in this analysis, we have included the data of 193 patients. If we decide to continue on with the study, the estimated sample sizes for the two-sample comparison of survivor functions, evaluated with the use of the Freedman method, compared with the log-rank test, with a level of alpha = 0.050 (two-sided), an estimated 5-year survival of 0.80 in one group and 0.75 in another, and a power of 0.80, would end up in a study group with 2186 patients, resulting in an HR of 1.289. If the estimated 5-year survival would be, respectively, 80% and 70%, there would be a need for 592 patients, with an HR of 1.598. The second estimation was actually closer to the obtained results.

## 4. Discussion

Our study demonstrates that there is a potential for the inclusion of an automated CAC score assessment and automated cardiac age assessment in the perioperative assessment in patients that are accepted for lung cancer surgery. The obtained results revealed typical prognosticators of complications in surgical lung cancer patients like the VATS approach and AACCI. In multivariable analysis, cardiac age was a potential prognosticator of the cardiovascular and pulmonary complications rate (95%CI −0.007–0.203, *p* = 0.066, beta coefficient 0.098). Moreover, coronary age (0–69 years of age vs. 70+ years of age) was also a potential prognosticator of OS (HR = 1.678, 95%CI 0.847–3.292 *p* = 0.138). Survival curves were separated enough and post hoc study sample calculation revealed that the continuation of the study would lead to potentially positive results. This is why the authors do not find this trial to be a negative one. Oppositely, in our opinion, it is an important step prior to its continuation. Despite analyses of huge datasets [9,10] proving that the CAC score is an important prognosticator in oncological patients and in smokers, scholars must find a need to validate these findings in a population of patients treated surgically due to NSCLC.

Our results are similar to results obtained by other teams. In a study by Osawa et al., a retrospective analysis of 309 patients that were operated on due to NSCLC revealed that the CAC score is a prognosticator of major adverse cardiovascular events (MACE), but has a relatively moderate effect (HR 1.18 95%CI 1.10–1.25 *p* < 0.01) [11]. When different categorical criteria in an analysis of data from 195 patients were applied, it was possible to reveal that a severe CAC score compared to an absent CAC score was related to a worse OS (HR 1.73 95%CI 1.03–2.88 *p* = 0.03) [12]. The power of the CAC score as a prognosticator was similar in a study by Shipe et al. and in our cohort. The findings obtained in the surgical series are also confirmed in different treatment protocols. Koutroumpakis et al. report that the CAC score sufficiently predicted MACE and OS (HR 1.64, 95%CI 1.11–2.44, *p* = 0.013) [13].

There are different ways of assessing the CAC score. It is not surprising that the automated CAC score is gaining scientific interest and clinical validation due to the easiness of the method and potentially important findings. Other teams also use automated systems managed by one scientist. Osawa et al. analyzed data with the Toshiba system [11]. On the other hand, some utilize a cross-verified method, yet also base this on the crude data also obtained from the Siemens platform (Syngo.via, Siemens Healthcare, Malvern, PA, USA) [13]. Other automated systems, labeled sometimes as artificial intelligence (AI) systems, are also used (Alienware Area 51 R6 equipped with Dual NVIDIA GeForce RTX 2080 OC graphics) [14]. Previously, studies have been undertaken over the non-automated evaluation of CAC scores on a 13-grade scale [7].

A number of studies have demonstrated the ability of AI to match the diagnostic accuracy of radiologists for CAC scores, while reducing the time required for evaluation [15,16,17]. In a study by Henriksson et al., they showed an excellent correlation and agreement between syngo.Via VB10A, Siemens Healthineers AI software and semi-automatic evaluation for the CAC score, the number of lesions, and lesion location [18]. In an international multicenter study, a deep learning system was used to quantify coronary plaque volumes from coronary CT angiography in 2803 patients and established age- and sex-specific distributions [19]. The AI-based plaque measurements revealed that plaque volumes increased with age and were higher in male patients. Patients with plaque volumes in the ≥75th percentile had a significantly higher risk of myocardial infarction [19]. Also, a retrospective analysis of the ADVANCE registry showed AI’s value in predicting clinical outcomes [20]. Differences in study design, inclusion, imaging protocols, reconstruction methods, and quantitative assessment make comparisons between studies with AI difficult. Nevertheless, the results for the CAC score and risk stratification using AI are promising. In our study, all of the patients had their CAC score assessed on PET CT. This examination is being undertaken in most of the patients that are due to undergo NSCLC resection. This approach is also shared by other researchers [12]. As there is only one scanner between the 2,000,000 inhabitants of the Pomeranian region of Poland, all of the examinations were performed according to one protocol, which strengthens the methodology of the current study. Another way of obtaining the data for CAC score assessment is the evaluation of data gathered in screening programs [7,14,21].

Study limitations: This study has several limitations. It is a retrospective analysis of a single-center study, which limits the potential to generalize the results. The CAC score was performed in a software environment without other systems’ validation. This may result in information biases. The data concerning the detailed history of CAD diagnostic procedures—ECG stress test and cardiac catheterization—were not aggregated, which could have altered the exact CAD diagnosis rate in study participants. Moreover, the data concerning the lipid profile, diabetes, family history, and other factors influencing CAD risk were not retrieved. Heart failure is a factor disqualifying most of the patients from lung cancer surgery, but the values of the left ventricle ejection fraction were not gathered systematically in the database and, due to missing values, were not included in the final analysis. The analysis of overall survival instead of cancer-related survival and MACE somehow limits the scientific meaning of the current study. Another limitation is the lack of aggregation of data typical for endpoints in oncological and cardiological survival analyses such as cancer-related survival and major adverse cardiovascular events. The study does not account for key factors that could influence outcomes, such as patients’ lipid profiles, diabetes status, and family history of coronary artery disease, which may lead to information biases.

One may argue whether the fact that the CAC score and coronary age were calculated automatically is a study limitation. We consider it a strength. There are some clinical data that are being accumulated by the means of advanced radiological software which lack adequate validation and an assessment of their meaning in everyday practice. To our understanding, automated CAC score assessment, as well as automated coronary age assessment, deserve thorough assessment in different clinical scenarios. Coronary age may be a prognosticator of OS in patients operated on due to NSCLC.

Another study limitation is the lack of information about adjuvant therapy, which may have slightly altered the OS in our patients.

The huge potential and rapid growth of user-friendly tools utilizing AI protocols has become widespread in different branches of medicine. In the opinion of the authors, it may be deemed unethical not to utilize those data for validation studies, for the sake of our patients in the future. AI should not be labeled as “meaningless”, “doubtful”, or “dangerous”. AI mechanisms of machine learning provide new tools that may add information concerning cachexia, emphysema, COPD, muscle strength, and others. These data are currently aggregated but require the implementation of adequate software to boost the power of those potentially important factors. The authors enthusiastically await current and future groundbreaking studies in the field, and contribute to the medical field with this paper. This approach requires validation studies. We believe it is not only required to conduct adequately powered trials, but to also compare different platforms that enable automated CAC score assessment.

In conclusion, the authors state that automated CAC score assessment may be a potential and clinically meaningful prognosticator of both postoperative complications and ultimately OS in patients that are operated on due to NSCLC.

## Figures and Tables

**Table 1:** Patient characteristics of the whole population. SD, standard deviation; n/a, non applicable; FEV1, forced expiratory volume; FEV1%, percent of the predicted value of forced expiratory volume; FVC, forced vital capacity; FVC%, percent of predicted value of forced vital capacity; VATS, video-assisted thoracic surgery.

Clinical Feature	Mean	SD
	176 (93%)	
Age	67.4	6.9
FEV1%	87.7	21.0
FVC%	98.4%	21.9
VATS	108 (56.0%)	n/a
Segmentectomy and wedge resection	25 (13.0%)	n/a
Lobectomy	159 (82.3%)	n/a
Pneumonectomy	9 (4.7%)	n/a
pIA	66 (33.2%)	
pIB	32 (16.6%)	
pIIA	13 (6.7%)	
pIIB	33 (17.1%)	
pIIIA	37 (19.2%)	
pIIIIB	12 (6.2%)	
Squamous cell carcinoma	69 (35.8%)	
Adenocarcinoma	103 (53.4%)	
Small cell lung cancer	6 (3.1%)	
Carcinoid	2 (1.0%)	
Other histologies	13 (6.7%)	
Charlson Comorbidity Index	1.3	1.4
Age-Adjusted Charlson Comorbidity Index	3.6	1.6
Preoperative diagnosis of coronary artery disease	18 (9.3%)	

**Table 2:** CAC values of coronary arteries in the evaluated population. CAC, coronary artery cardiac score; LM, left main coronary artery; LAD, left anterior descending coronary artery; CX, left circumflex coronary artery; RCA, right coronary artery.

Clinical Feature	Mean	SD
CAC score	519.0	800.1
LM score	73.2	161.7
LAD score	157.8	263.1
CX score	110.2	272.5
RCA score	177.8	415.3
Coronary age		
0–30	36 (18.7%)	
30–39	0 (0.0%)	
40–44	2 (1.0%)	
45–49	7 (3.6%)	
50–54	9 (4.7%)	
55–59	20 (10.4%)	
60–64	13 (6.7%)	
65–69	13 (6.7%)	
70–74	8 (4.1%)	
75–89	0 (0.0%)	
90+	85 (44.0%)	

**Table 3:** Postoperative complications with attributed mean CAC score.

	Proportion	Mean CAC Score in Patients with Complication (SD)	Mean CAC Score in Patients Without Complication (SD)	*p* Value
General complications rate	85/193 (44.0%)	619 (827)	528 (861)	0.460
Cardiovascular and pulmonary complications rate	60/193 (31.1%)	656 (856)	528 (841)	0.332
Cardiovascular complications rate	27/193 (14.0%)	474 (831)	583 (849)	0.535

**Table 4:** Multivariable models testing different sets of prognosticators influencing the complication rates. FEV1%, forced vital capacity in 1 s % of reference value; FVC%, forced vital capacity % of reference value; VATS, video-assisted thoracic surgery; CAC, coronary artery score; AACCI, Age-adjusted Charlson Comorbidity index; LM, left main coronary artery; LAD, left anterior descending coronary artery; CX, left circumflex coronary artery; RCA, right coronary artery.

(**a**)					
**Cardiovascular and Pulmonary Complications**					
	**Coefficient**	**Std. Err.**	***p* Value**	**95% Conf.**	**Interval**
FEV1%	0.0021488	0.0025525	0.401	−0.0028871	0.0071847
FVC%	−0.0017888	0.0024449	0.465	−0.0066124	0.0030348
VATS	−0.0933078	0.0635414	0.144	−0.2186713	0.0320556
Lobe_segm_pneum	−0.1998688	0.0462291	0.000	−0.291076	−0.1086616
CAC score	2.64 × 10^−6^	0.0000448	0.953	−0.0000858	0.0000911
Cardiac age	0.014442	0.0124605	0.248	−0.0101418	0.0390258
AACCI	0.0246801	0.0207862	0.237	−0.0163298	0.06569
_cons	0.621076	0.2513949	0.014	0.1250888	1.117063
(**b**)					
**Cardiovascular Complications**					
	**Coefficient**	**Std. Err.**	***p* Value**	**95% Conf.**	**Interval**
FEV1%	−0.0012335	0.0019964	0.537	−0.0051722	0.0027051
FVC%	0.0020373	0.0019123	0.288	−0.0017354	0.0058099
VATS	0.0523474	0.0496638	0.293	−0.0456328	0.1503276
Lobe_segm_pneum	−0.0863515	0.0360439	0.018	−0.1574615	−0.0152415
CAC score	−0.0000231	0.0000351	0.511	−0.0000923	0.0000461
Cardiac age	0.0044426	0.0095494	0.642	−0.0143972	0.0232823
_cons	0.0141421	0.1914593	0.941	−0.3635823	0.3918664
(**c**)					
**General Complications**					
	**Coefficient**	**Std. Err.**	***p* Value**	**95% Conf.**	**Interval**
FEV1%	0.0013758	0.002628	0.601	−0.0038089	0.0065606
FVC%	0.0004877	0.0025173	0.847	−0.0044786	0.0054539
VATS	−0.117375	0.0653764	0.074	−0.2463542	0.0116042
Lobe_segm_pneum	−0.2832119	0.0474475	0.000	−0.3768197	−0.1896041
CAC score	7.05 × 10^−6^	0.0000462	0.879	−0.000084	0.0000981
Cardiac age	0.0105531	0.0125707	0.402	−0.0142472	0.0353534
_cons	0.8756683	0.2520333	0.001	0.3784394	1.372897
(**d**)					
**General Complications**					
	**Coefficient**	**Std. Err.**	***p* Value**	**95% Conf.**	**Interval**
CAC score	−0.0001937	0.0003578	0.589	−0.0008995	0.0005121
LM	0	(omitted)			
LAD	0.0003449	0.0004573	0.452	−0.0005573	0.0012471
CX	0.0000875	0.0004186	0.835	−0.0007383	0.0009133
RCA	0.0002707	0.000367	0.462	−0.0004532	0.0009947
_cons	0.4268289	0.044	0.000	0.3400288	0.513629
(**e**)					
**Cardiovascular Complications**					
	**Coefficient**	**Std. Err.**	***p* Value**	**95% Conf.**	**Interval**
CAC score	−0.0003934	0.0002493	0.116	−0.0008852	0.0000985
LM	0	(omitted)			
LAD	0.0005675	0.0003187	0.077	−0.0000612	0.0011962
CX	0.0003486	0.0002917	0.234	−0.0002269	0.000924
RCA	0.0003496	0.0002557	0.173	−0.0001549	0.0008541
_cons	0.1515291	0.0306602	0.000	0.0910447	0.2120135
(**f**)					
**Cardiovascular and Pulmonary Complications**					
	**Coefficient**	**Std. Err.**	***p* Value**	**95% Conf.**	**Interval**
CAC score	−0.0003877	0.0003302	0.242	−0.0010391	0.0002638
LM	0	(omitted)			
LAD	0.0006158	0.0004221	0.146	−0.0002169	0.0014485
CX	0.0002392	0.0003864	0.537	−0.000523	0.0010014
RCA	0.0005019	0.0003387	0.140	−0.0001663	0.0011701
_cons	0.2942409	0.0406115	0.000	0.2141253	0.3743564

## Data Availability

Anonymized data are available on request.
